# Early Adaptive Schemas and Sexual Wellbeing in Women: Exploring Differences in Menopausal Status

**DOI:** 10.1007/s41042-023-00100-x

**Published:** 2023-05-16

**Authors:** Andrew Allen, Colleen Tully-Wilson

**Affiliations:** School of Health, University of the Sunshine Coast, 90 Sippy Downs Drive, Sippy Downs, Queensland 4556 Australia

**Keywords:** Early adaptive schema, Menopause, Sexual wellbeing, Sexual satisfaction, Sexual functioning, Positive psychology

## Abstract

There is limited research concerning the relationship between early adaptive schema, from Young’s Schema Theory, and women’s sexual wellbeing. Schema Theory posits that early adaptive schema start forming in early childhood from core emotional needs being met, and positively influence individuals’ concept of self, relationships with others, and their behaviours. Building on this theory, the current study explored the relationship of early adaptive schema and adult women’s sexual wellbeing at pre-, peri-, and post-menopause. Four hundred and sixty-seven women, mostly partnered and heterosexual, from over ten countries, participated in an online survey measuring relationships between early adaptive schema and sexual wellbeing, measured by sexual functioning and sexual satisfaction. The strength of association of early adaptive schema and sexual well-being were evaluated in addition to known predictors. The results showed higher early adaptive schema were associated with higher sexual wellbeing, measured by sexual satisfaction and sexual functioning,with medium-to-large effect sizes, at pre and peri-menopause, and produced a non-significant result for post-menopause. The association of early adaptive schema continued after known factors were accounted for. The results support the use of early adaptive schema to promote sexual wellbeing for women at pre- and peri-menopause.

The relevance and acceptance of adult women’s sexual wellbeing as a state differing from the absence of sexual dysfunction or disease, has been promoted by practitioners and researchers (Garrett & Vaisey, 2020; Lorimer et al., 2019), and reflected in a statement by the World Health Organisation in 2017 acknowledging sexual health as “a state of physical, emotional, mental, and social well-being in relation to sexuality”. Sexual activity is integral to both men’s and women’s wellbeing across the lifespan (Stephenson & Meston, 2015). Despite the links with wellbeing, research on sexual health has traditionally focused on sexually transmitted infections (Burns et al., 2016), sexual violence and abuse (Abrahams et al., 2014; Barnum & Perrone-McGovern, 2017), sexual dysfunction (Buster, 2013; Weinberger et al., 2019), and disease or medical interventions (Stanton et al., 2018; Syme et al., 2013). There is consequently a gap in knowledge of the factors that contribute to positive sexual wellbeing as an aspect of overall wellbeing, rather than a lack of sexual dysfunction exclusively (Graf & Patrick, 2014; Martin & Woodgate, 2020).

Stephenson and Meston (2015), when exploring the relationship of sexual wellbeing and life satisfaction, used a definition of sexual wellbeing that included a subjective sexual satisfaction element and sexual distress, encompassing both psychosocial and physiological aspects (Stephenson & Meston, 2015). Similarly, Lorimer et al. (2019) defined sexual wellbeing as combining a subjective individually experienced component and an objective element involving physical functioning and social structures that enable opportunities and sexual freedom (Lorimer et al., 2019). In support of this previous research and without an agreed generic definition, this study incorporates the separate elements of sexual satisfaction and sexual functioning as being representative of sexual wellbeing.

## Predictors of Sexual Wellbeing

There is wide support for a biopsychosocial approach to sexual wellbeing, in both identifying causal factors for positive sexual health and in the treatment of sexual dysfunctions (Caruso et al., 2016; Delamater, 2012; Emmerlink et al., 2016). Acknowledging the complexity of sexual wellbeing, this approach incorporates biological aspects of sexual functioning, the idiosyncratic subjectivity of sexual satisfaction, the systemic effects of physical and mental wellbeing, and how individual cognitions, interpersonal relationships, and wider social and cultural factors can influence sexual health (Arenella & Steffen, 2021; Delamater & Koepsel, 2015; Horley & Clarke, 2016; Simon et al., 2018).

In support of this approach, predictors for women’s sexual functioning include physical and psychological wellbeing, their relationship satisfaction, and a woman’s partner’s sexual functioning (Peixoto & Nobre, 2020; Scavello et al., 2019). Both physical and psychological conditions affect womens’ quality of life, and predict their sexual wellbeing, at all stages of life (McCabe et al., 2020; Simon et al., 2018). Simon et al. (2018) also explain how a partner’s sexual functioning can predict a woman’s sexual functioning due to the complex interplay of physiological and interpersonal changes, though research in this area is limited for post-menopausal women (Simon et al., 2018).

Changes in women’s hormones in post-menopause and the subsequent physiological effects on sexual functioning are well researched (Heidari et al., 2019; Simon et al, 2018). The lowering levels of sex steroids have neurobiological consequences that affect sexual wellbeing, including cognitive functions and dopaminergic transmission related to sexual behaviour (Scavello et al., 2019). Hormone replacement treatment (HRT), involving estrogen and/or androgen replacement, has been found to have a positive effect on sexual functioning in post-menopausal women (Scavello et al., 2019; Simon et al., 2018).

Acknowledging the subjectivity of sexual satisfaction as a measure of sexual wellbeing, and the influence and comorbidity of individual, relationship, and social influences, Rausch and Rettenberger (2021), in their systemic review of women’s sexual satisfaction predictors, identified sexual functioning and behaviours, and mental and physical health, as commonly accepted factors associated with sexual satisfaction (Rausch & Rettenberger, 2021). In later stages of women’s life, these factors remain similar, with physical health, sexual functioning, and negative affect found to be individual factors associated with sexual wellbeing (Arenella & Steffen, 2021).

Thoughts, beliefs, and emotions about the self and the sexual partner have been found to influence sexual functioning (Huberman et al., 2021; Oliveira & Nobre, 2013) and sexual satisfaction (Rausch & Rettenberger, 2021). For example, Emmerink et al. (2016), in their study of young heterosexual adults, found that traditional gender-related negative and positive sexual attitudes impacted women’s sexual autonomy, sexual body esteem, and sex avoidance (Emmerink et al., 2016). Similarly, Peixoto and Nobre (2020) studied psychological factors that predict sexual functioning in both lesbian and heterosexual female groups (mean age 26 years) and found that positive affect and less negative thoughts (failure, disengagement) significantly predicted women’s sexual functioning (Peixoto & Nobre, 2020). Segnini & Kukkonen (2018), when reviewing therapy for sexual arousal disorders, advocate for a biopsychosexual model of treatment that identifies the interrelated factors that form barriers to sexual wellbeing, and tailoring treatment accordingly. According to Segnini and Kukkonen (2018), physical difficulties in sexual functioning are best addressed through biological treatments such as pharmaceutical approaches, and psychological and cognitive barriers to sexual health, for example, those relating to an individual’s automatic thoughts and core beliefs, are best addressed through psychotherapies (Segnini & Kukkonen, 2018). A psychotherapy that acknowledges patterns and interactions of cognitive beliefs is Young’s Schema Therapy (Young et al., 2003).

## Schema Theory

Schema Theory was developed by Jeffrey Young for people with personality disorders and complex psychosocial symptoms (Young et al., 2003). Schemas are conceptualised as persistent and pervasive patterns of thoughts, beliefs, emotions, and behaviours, used to guide attention, process information, create knowledge, and anticipate future events (Young et al., 2003). Young (2003) described how schema form in early childhood and adolescence, depending on whether emotional needs such as attachment, autonomy, and emotional expression are satisfied, leading to early adaptive schema;, or impeded, leading to early maladaptive schemas (Bach et al., 2018; Oliveira & Nobre, 2013; Young et al., 2003). Building on the work of Bowlby’s (1982) Attachment Theory and the importance of attachment to key carers for a child’s development, Young et al. (2003) extended an individual’s schema development influences to childhood trauma, the individual’s temperament, culture, and family relationships. Schema can then be applied, and later arise, in different contexts including adult roles of parenting, eating behaviours, and sexual self-schemas (Bijkeboer & Boo, 2009; Bishop et al., 2022;Huberman et al., 2021).

While research has identified associations between early maladaptive schemas and sexual dysfunction (Alimoradi et al., 2022; Oliveira & Nobre, 2013), victimization in intimate partner violence (Hassija et al., 2018), interpersonal problems (Janovsky et al., 2020), and risky sexual behaviour (Roemmele & Messman-Moore, 2011), less is known about early adaptive schema (Lockwood & Perris, 2012). Early schema’s lifetime progress is unknown, however, it is thought that they remain stable and are triggered by psychosocial stressors (Videler et al., 2020). Videler et al. (2020) propose that early adaptive schema are expressed strongly in middle-age due to the accumulation of knowledge and their usefulness as contributors to healthy coping strategies (van Donzel et al., 2021; Videler et al., 2020). Early adaptive schema influence an individuals’ self-perception alongside their interpersonal relationships, interpretation of the world, and patterns of automatic affect and behaviour (Louis et al., 2018b). They are associated with independent functioning and successful interpersonal relationships (Lockwood & Perris, 2012). For example, a child who experiences their emotional needs met, may develop higher levels of the early adaptive schema of Emotional Fulfilment and Self-Care. These schema enable them to choose healthy adult relationships where they are able to cope with distress by voicing their emotional needs, leading to higher relationship and sexual satisfaction (Sundgren & Allen, 2022). Both early maladaptive schema and early adaptive schema can be present simultaneously but are comprised of distinct constructs (Lockwood & Perris, 2012).

Research supports the association of low levels of early maladaptive schemas and women’s sexual functioning and sexual satisfaction (Oliveira & Nobre, 2013; Peixoto & Nobre, 2020). However, because schema therapy was originally developed for participants with borderline personality disorder, the majority of this research has been completed in populations with adverse childhood events (Pilkington et al., 2020). Andersen and Cyranowski (1998) conceptualized women’s sexual self-schemas as being developed through childhood and adult experiences, including sexual experiences and their broader views of emotional and intimate relationships, that are then activated in romantic attachment and sexual contexts. These sexual schemas are dynamic and complex, building on positive and negative interpersonal experiences and self-views that influence sexual thoughts, emotions, and response outcomes (Cyranowski & Andersen, 1998).

Despite the complexity of women’s sexual wellbeing and the influence of both positive and negative experiences, there is little research investigating the role of early adaptive schema on women’s sexual wellbeing and whether their relationship changes at different stages of women’s reproductive states. This is important because there are different psychosocial life stressors, in addition to the physiological and psychological effect of hormonal and age-related changes, reflected by the menopausal life stages of pre-, peri-, and post-menopause (Nappi, 2007). Pre-menopause marks the start of a female’s reproductive ability where menstrual cycles are regular and post-menopause is defined as a minimum of twelve months post reproductive function being lost (Burger, 2013; Santoro et al., 2021). The transition between pre- and post-menopause, labelled perimenopause, is highly variable and commences with menstrual irregularities associated with fluctuating and declining sex hormone levels (Burger, 2013). Scientific consensus now identifies menopausal stages as mutually exclusive and categorized using objective hormonal test data, acknowledging that the symptoms of ovary lifespan changes are similar for all women, the stages can be affected by multiple factors including body mass index, HRT, prior hysterectomy, and abnormal uterine anatomy (Ambikairajah et al., 2022).

## Research Rationale

Positive clinical psychology posits that interventions, such as the identification and use of early adaptive schema, can increase positive affect and cognitions and successfully address psychopathology, forming an alternative or complementary therapy to traditional treatments that focus on decreasing negative affect, thoughts, and behaviours (Wood & Tarrier, 2010), and enable protection from mental ill-health (Louis et al., 2018b). The results of this research can inform psychotherapy for women with clinical sexual dysfunctions and support positive sexual wellbeing.

This research aims to explore the relationship between early adaptive schema (Louis et al, 2018a) measured through the Young’s Positive Schema Questionnaire (YPSQ) and sexual wellbeing, measured by sexual satisfaction (Stulhofer et al., 2009) and sexual functioning (Rosen et al., 2000), for women at different stages of menopause. In addition, the study analyses the relationship of early adaptive schema levels with sexual functioning and sexual satisfaction in addition to known predictors. Acknowledging the complexity and correlations between known predictors, the researchers chose predictors that consistently emerged from previous studies and encompass a biopsychosocial approach.

The study had three hypotheses: firstly, that higher levels of early adaptive schema will be significantly associated with higher sexual functioning and sexual satisfaction for women at pre, peri, and post-menopause; secondly, the association of early adaptive schema on levels of sexual satisfaction and sexual functioning will vary across pre-, peri-, and post-menstrual women; and thirdly, that the results will show differing relationships between early adaptive schema domains and sexual wellbeing (sexual functioning and sexual satisfaction), based on women’s menopausal status.

## Method

### Participants

A total of 467 women, recruited through online social media and snowballing, participated in the study, encompassing the three subgroups of 284 pre-, 136 peri-, and 47 post-menopausal women. Participants self-selected their menopause status. Due to the complexity of peri-menopause, and the reliance on biological testing, participants were given definitions of pre-, peri-, and post-menopause based on the menstruation symptoms and asked if they identified with this stage. These definitions included: Pre-menopausal – regular menstrual bleeding in the last 12 months (unless disrupted by pregnancy, medication, or illness); Peri-menopausal (irregular menstrual bleeding or an absence of menstrual bleeding for more than 3 months but less than 12 months); and Post-menopausal – surgical intervention or no menstrual bleeding for 12 months or more. The demographic characteristics of the groups are shown in Table 1. The European countries were combined because of low participant numbers.

**Table 1:** Demographics for Pre-, Peri-, and Post-menopause groups

	Pre-menopause *n* = 284	Peri-menopause *n* = 136	Post-menopause *n* = 47
Age (*SD*), and Range (years)	30.20 (7.09), 18—51	36.77 (7.90), 21—52	54.26 (10.15), 33—77
Relationship Status	partner/married—65.9% single^a^—32.8%	partner/married – 72.3% single^a^—27.8	partner/married – 80.8% single^a^—17.0%
Current sexual partner	70.8%	60.6%	85.1%
Sexual orientation	heterosexual—60.2% bisexual—14.4%, gay/lesbian—18.0% pansexual – 3.2% asexual – 0%	heterosexual – 71.5% bisexual – 8.8%, gay/lesbian – 16.6% pansexual – 2.9% asexual – 0%	heterosexual -78.7%, bisexual – 0% gay/lesbian – 17.1% pansexual – 2.1% asexual – 2.1%
Education	15 + years—65.8% 13 – 14 years—18.7% 8 – 12 years—14.8% < 8 years – 0%	15 + years – 67.2% 13 – 14 years – 16.1% 8 – 12 years – 16.1% < 8 years – 0.7%	15 + years – 66.0% 13 – 14 years – 14.9% 8 – 12 years – 19.1% < 8 years – 0%
Country/Area of Residence	America (US) 38.7% Australia 24.6% Canada 10.9% Mexico 9.5% Europe^b^ 12.0%	America (US) 28.5% Australia 19.7% Canada 10.9% Mexico 18.2% Europe^b^ 12.4%	America (US) 34.0% Australia 46.8% Canada 4.4% Mexico 0% Europe ^b^ 6.4%
	Other^c^ 4.3%	Other^c^ 10.3%	Other^c^ 8.4%

^a^includes divorced and widowed participants; ^b^Europe includes Austria, Belgium, Czech Republic, France, Germany, Ireland, Italy, Norway, Scotland, Spain, Sweden, and United Kingdom; ^c^Other includes China, Japan, New Zealand, and Russia

### Materials

Data was gathered via an anonymous online survey comprised of informed consent, demographic questions, questions regarding menopause status, and standardised measures of female sexual functioning, sexual satisfaction, and early adaptive schema. The questionnaire was in English. Participants were not filtered based on their English language proficiency. Participants were asked if they had been diagnosed with a current medical physiological or psychiatric condition by a health professional.

### Measures

The Female Sexual Functioning Index (FSFI; Rosen et al., 2000) measures sexual dysfunction with a 19-item scale and 0 (or 1) to 5 Likert-type categories; a 0 response indicating no sexual activity and 1–5 responses varying from very low to very high or almost never/never to almost always/always depending on question context. The original English version was used (Rosen et al., 2000) comprising six sub-scales of desire, arousal, lubrication, orgasm, satisfaction, and pain. Using algorithms, the scales are collated so that each subscale has a maximum total of 6 and the total index, being an addition of the six subscales, ranging from 2 – 36 with higher scores representing better sexual functioning. The scale has been validated in clinical (Bartula & Sherman, 2015) and non-clinical populations (Kalmbach et al., 2015). Construct validity was established by Rosen et al. (2000). Test–retest reliability demonstrated good internal consistency (Cronbach alphas varying from 0.79 to 0.96 across the three groups).

The New Sexual Satisfaction Scale (NSSS; Stulhofer et al., 2009) is a 20-item measure of sexual satisfaction. The scale was based on five conceptual domains: sexual sensations, sexual presence/awareness, sexual exchange, emotional connection/closeness, and sexual activity, with analysis confirming a two-dimension structure of self-focus “ego-centered” and “partner- and sexual activity-centered”. Answers are measured with a 5-point Likert scale (1 = not at all satisfied to 5 = extremely satisfied) based on participants’ sexual activity in the previous six months. Higher scores represent greater sexual satisfaction. The scale is sexual-orientation and relationship-status generic and has been used in clinical and nonclinical studies (Štulhofer et al., 2010). The scale showed high internal consistency across the three groups (Cronbach’s Alphas of 0.93 to 0.98).

The Young Positive Schema Questionnaire (YPSQ; Louis et al., 2018a) was developed to measure adults’ early adaptive schema. The YPSQ is a 56-item, 14-factor scale using 6-point Likert-type scores ranging from 1 (*completely untrue of me*) to 6 (*describes me perfectly*). Louis et al. (2018a)’s study, using Western and Eastern countries’ samples, demonstrated that the YPSQ held good factorial validity (convergent, divergent, and construct). Additional validity was demonstrated in research by Louis et al. (2018c) between the YPSQ and subscales of the Positive Parenting Schema Inventory. The scale showed high internal reliability with Cronbach Alphas from 0.93 to 0.98 for the three groups. In 2020, Louis et al. identified and tested a four-factor model of higher order early adaptive schema, representing four core emotional needs (Louis et al., 2020). The four higher order domains and associated early adaptive schema are outlined in Table 2.

**Table 2:** YPSQ four higher order early adaptive schema domains and the individual factors

YPSQ higher order domain	Early adaptive schema
Realistic Standards and Reciprocity	Realistic Expectations Empathatic Consideration Self-Directness Self-Compassion Basic Health/and Safety/Optimism
Connection and Acceptance	Emotional Fulfilment Social Belonging Emotional Openness and Spontaneity Healthy Self-Interest/Self-Care
Healthy Autonomy and Performance	Healthy Self-Reliance/Competence Healthy Boundaries and Developed Self Stable Attachment
Realistic Limits	Healthy Self-Control Success

The known variable data for sexual functioning and sexual satisfaction was collected via the survey. The presence of medical/psychological problems was measured by two ‘yes/no/maybe’ questions: “Do you have any current medical physiological conditions that have been diagnosed by a health professional” and “Do you have any current psychiatric conditions that have been diagnosed by a health professional?” These two questions were combined: a response “no” for both questions was considered “no” and the remaining responses were considered “yes”. A question was asked of all respondents to identify the presence of a partner, and, if present, their sexual functioning: “Is your partner currently diagnosed with a sexual function disorder or taking medication that restricts their sexual functioning?”. Answers of ‘yes or maybe/unsure’ were combined to indicate partner sexual dysfunction. The use of HRT applied only to those indicating they were post-menopausal. The question “Do you currently take hormone therapy (e.g., estrogen, androgen, testosterone) for postmenopausal sexual functioning or to alleviate menopausal symptoms?”. Answer categories were ‘yes, no, or unsure.’ ‘Yes and unsure’ answers were combined and compared with a 'no’ response.

### Procedure

The survey was developed using Qualtrics software (Qualtrics, Provo, UT). After the study gained ethical approval from a university ethics board (number S201486), participants were recruited through social media (Facebook and Reddit sites), and snowballing. Participants were offered the opportunity to win one of four AUD$50.00 Amazon vouchers. The sample was collected between January and August 2021. Participants were informed of the purpose and anonymity of the study. Participants were divided into three groups based on their self-reported menopause status: pre-menopausal (*n* = 284), peri-menopausal (*n* = 136), and post-menopausal (*n* = 47). G*Power analysis (Faul et al., 2009) for a linear regression, R-squared, deviation from zero, with four variables, indicated a minimum of 53 participants for each group (alpha of 0.05, power 0.80, effect size of 0.25). The post-menopause group comprised of 47 responses, resulting in low power for significant findings.

### Data Analysis

The data were analysed using the software IBM SPSS Statistics for Windows, Version 27.0 (IBM Corp, 2020). Multiple regression analyses (MRAs) were used to test whether participants with higher levels of early adaptive schema (predictor variables) would report higher levels of sexual wellbeing as measured by sexual functioning and sexual satisfaction (outcome variables). The associations of early adaptive schema was explored through two MRAs for each outcome variable: MRA1 used the early adaptive schema’s four domains to evaluate their individual relationship with sexual functioning and sexual satisfaction, and MRA2 used two-step hierarchical MRAs to identify if the total for early adaptive schema (the four domains combined), contributed to the the outcome variable totals, in addition to predictor variables identified in previous research.

Variables that related to sexual functioning and sexual satisfaction, used for MRA2, differed for both outcome variables. For sexual functioning in the pre- and peri-menopause groups, the previously researched predictors used were physical/psychological disorders and partner’s sexual functioning status. With limited power due to low responses and less research on sexual functioning predictors in the post-menopause group, the MRA2 for post-menopausal women utilized physical/psychological disorders and the use of HRT as the known predictors. The number of known predictors for the post-menopause group was limited in the analysis due to the low number of participants in this group. The use of HRT was included, as opposed to partner’s sexual function, because it had a strong significant correlation with the outcome of sexual functioning (refer to supplementary material). For sexual satisfaction, the previously researched predictors used in Step 1 of MRA2 were sexual functioning and physical and psychological disorders for each menopausal group, with the total level of early adaptive schema added at Step 2. All known predictors were added as a block in Step 1 for the MRA2s. Refer to Table 3. Effect sizes were calculated according to Cohen’s (1962) criteria and underly the use of large, medium, and small effects as descriptors.

**Table 3:** MRA 2: two-step hierarchical regression predictor variables for sexual functioning and sexual satisfaction

	Pre-menopause *n* = 284	Peri-menopause *n* = 136	Post-menopause *n* = 47
Sexual Functioning Step 1 (block)	Physical and Psychological Disorders, Partner’s Sexual Functioning	Physical and Psychological Disorders, Partner’s Sexual Functioning	Physical and Psychological Disorders, Use of HRT^a^
Step 2	Total Early Adaptive Schema	Total Early Adaptive Schema	Total Early Adaptive Schema
Sexual Satisfaction Step 1 (block)	Physical and Psychological Disorders, Sexual Functioning	Physical and Psychological Disorders, Sexual Functioning	Physical and Psychological Disorders, Sexual Functioning
Step 2	Total Early Adaptive Schema	Total Early Adaptive Schema	Total Early Adaptive Schema

^a^Hormone Replacement Therapy (HRT)

## Results

### Assumption Testing and Data Cleaning

Incomplete responses, duplicates, and spurious submissions were identified and removed. In total, 9 participants were removed from the post-menopause group, 28 from the peri-menopause group, and 57 from the pre-menopause group. A small percentage of responses (three responses of one YPSQ domain in the pre-menopause group, and 3 responses of the partner index of sexual satisfaction in the peri-menopause group) were identified as having totals of 4 or more standard deviations from the mean and were windsorized to 3 standard deviations from the mean to prevent the results being skewed (Tabachnick & Fidell, 2014). Data for Sexual Functioning and Sexual Satisfaction was negatively skewed in all three groups, however, transformations were not used to preserve data integrity (Tabachnick & Fidell, 2014). Normality was assumed for the pre- and peri-menopause groups due to the respective group sizes (Wilcox, 2001). The post-menopause group was bootstrapped due to the small sample size (Field, 2015). Multicollinearity was evident when using the four YPSQ Domains to identify Sexual Satisfaction and Sexual Functioning, however, since the analyses’ aim was to determine relationships, this was accepted (Tabachnick & Fidell, 2014). In the two-step MRAs, the YPSQ total score was used due to the high correlation between variables. All two-step models had acceptable multicollinearity as shown by the correlation analyses and VIF levels. Multivariate outliers with high Mahalanobis distances and exceeding the critical χ^2^ for *df* = 3 (at α = 0.001) of 16.27 and *df* = 4 (at α = 0.001) of 18.47 were deleted, except for one case in each pre-menopause and peri-menopause groups that were retained due to a low leverage value and acceptable Cook’s Distance. All cases between 2.5 and 3 standard deviations were assessed individually and, based on their number being less than two percent of total cases, were retained.

### Sexual Functioning

To test the hypothesis that higher levels of early adaptive schema would be significantly associated with higher levels of sexual functioning for each group, a MRA was performed entering the four YPSQ domains as a block of independent variables. Table 4 shows the means for each group and the variance accounted for by the total four domains. The sexual functioning mean of the pre-menopausal group was within the confidence interval of the mean of the control group used by Rosen et al. (2000) when developing the FSFI. The declining sexual functioning mean due to peri- and further still, post-menopause, were in line with previous research that identified older age as a risk factor for sexual dysfunctions (Arenella & Steffen, 2021). The influence of the YPSQ domains were significant (*p* =  < 0.001) in the pre-menopausal and peri-menopausal groups. Table 5 reports the unstandardised (*B*), and standardised regression coefficients (*β*), standard error (*SE*) and probability (*p*) for each variable. Correlations for the variables from the MRAs for each of the three groups, pre-, peri-, and post-menopause, are listed in Appendix A. The domain of Connection and Acceptance was a significant variable in the pre-menopausal group (*p* = 0.033).

**Table 4:** Sexual functioning and YPSQ domains

Groups	Mean (*SE*)	*R*^2^	Adj *R*^2^	*p*	Effect
Pre-menopause (*n* = 284)	26.53 (4.72)	0.16	0.15	< 0.001	Medium^a^
Peri-menopause (*n* = 136)	24.92 (4.29)	0.340	0.32.0	< 0.001	Large^a^
Post-menopause (*n* = 47) ^b^	21.38 (9.13)	0.10	0.01	0.361	NS

^a^Cohen (1962) effect sizes, ^b^bootstrapped, *NS* not significant

**Table 5:** MRA1 sexual functioning with four YPSQ domain variables

Groups	*B* [95% CI]	*SE B*	*β*	*p*
Pre-menopause *R*^2^ = 0.16, *F* (4,279) = 13.03, *p* < 0.001				
Constant	16.90 [14.16, 19.63]	1.39		< 0.001
Realistic Standards and Reciprocity	0.57 [–0.65, 1.78]	0.62	0.10	0.360
Connection and Acceptance	1.32 [0.11, 2.53]	0.62	0.23	0.033
Healthy Autonomy and Performance	–0.21 [–1.20, 0.77]	0.50	–0.04	0.669
Reasonable Limits	0.66 [–0.32, 1.63]	0.50	0.13	0.185
Peri-menopause *R*^2^ = 0.34, *F* (4, 131) = 16.88, *p* < 0.001				
Constant	11.87 [8.66, 15.08]	1.62		< 0.001
Realistic Standards and Reciprocity	1.24 [–1.01, 3.49]	1.14	0.24	0.278
Connection and Acceptance	1.00 [–1.45, 3.46]	1.24	0.19	0.421
Healthy Autonomy and Performance	1.43 [–0.68, 3.53]	1.06	0.27	0.182
Reasonable Limits	–0.53 [–2.11, 1.05]	0.80	–0.11	0.508
Post-menopause^a^ *R*^2^ = 0.10, *F* (4,42) = 1.12, *p* = 0.361				
Constant	22.49 [4.78, 40.20]	8.78		0.014
Realistic Standards and Reciprocity	–6.15 [–13.74, 1.43]	3.76	–0.46	0.109
Connection and Acceptance	4.11 [–1.14, 9.35]	2.60	0.36	0.122
Healthy Autonomy and Performance	4.00 [–2.29, 10.29]	3.12	0.38	0.206
Reasonable Limits	–2,49 [–7.76, 2.78]	2.61	–0.25	0.346

*CI* confidence interval. ^a^ Confidence intervals and standard errors for Post-menopause group based on 1000 bootstrap samples

To identify whether early adaptive schema would be associated with more variance than factors previously shown to predict sexual functioning, a two-step hierarchical MRA was performed. For pre- and peri-menopausal groups Step 1 entered physical and psychological disorders, and partner’s sexual functioning status, as a block, and the total YPSQ was added at Step 2. The total YPSQ was used because of the high correlation between the four domains (pre-menopause *r* = 0.744—0.840, peri-menopause: *r* = 0.838—0.916, post-menopause *r* = 0.617- 0.811), refer to Appendix A. Predictors of sexual functioning in the post-menopausal group, entered as a block at Step 1 were physical and psychological disorders, and the use of HRT. Step 2 added the YPSQ total. Refer to Table 6.

**Table 6:** MRA2 sexual functioning: two-step hierarchical multiple regression model

Groups		*B* [95% CI]	*SE B*	*β*	*p*
Pre-menopause	Step 1 *R*^2^ = 0.04 (*p* = 0.002)				
	Constant	22.51 [20.02, 24.99]	1.26		< 0.001
	Physical/Psychological Disorder	–0.36 [–1.65, 0.92]	0.65	–0.03	0.578
	Partner Sex Dysfunction	1.65 [0.69, 2.60]	0.48	0.20	< 0.001
	Step 2 Δ*R*^2^ = 0.14 (*p* < 0.001), *R*^2^ = 0.18 (*p* < 0.001)				
	Constant	13.08 [9.54, 16.62]	7.27		< 0.001
	Physical/Psychological Disorder	0.99 [–0.26, 2.24]	0.64	0.09	0.121
	Partner Sex Dysfunction	1.41 [0.53, 2.30]	0.45	0.17	0.002
	Total YPSQ	0.58 [0.41, 0.75]	0.08	0.39	< 0.001
Peri-menopause	Step 1 *R*^2^ = 0.00 (*p* = 0.836)				
	Constant	25.71 [22.96, 28.45]	1.39		< 0.001
	Physical/Psychological Disorder	0.49 [–2.29, 2.39]	1.18	0.00	0.967
	Partner Sex Dysfunction	–0.37 [–1.59, 0.85]	0.62	–0.05	0.552
	Step 2 Δ*R*^2^ = 0.34 (*p* < 0.001), *R*^2^ = 0.34 (*p* < 0.001)				
	Constant	13.94 [10.32, 17.56]	1.83		< 0.001
	Physical/Psychological Disorder	0.65 [–1.27, 2.57]	0.97	0.05	0.503
	Partner Sex Dysfunction	–0.94[–1.95, 0.07]	0.51	–0.13	0.067
	Total YPSQ	0.19 [0.15, 0.24]	0.02	0.59	< 0.001
Post-menopause^a^	Step 1 *R*^2^ = 0.15 (*p* = 0.028)				
	Constant	31.73 [21.94, 41.41]	5.06		< 0.001
	Physical/Psychological Disorder	0.554 [–4.48, 5.49]	2.51	0.03	0.824
	HRT	–7.29 [–12.46, –2.19]	2.58	–0.39	0.011
	Step 2 Δ*R*^2^ = 0.01 (*p* < 0.001), *R*^2^ = 0.16 (*p* = 0.055)				
	Constant	26.98 [8.49, 43.55]	8.77		0.004
	Physical/Psychological Disorder	–0.24 [–5.92, 5.39]	2.87	–0.01	0.936
	HRT	–7.77 [–12.85, –2.95]	2.54	–0.41	0.005
	Total YPSQ	0.38 [–0.59, 1.52]	0.54	0.12	0.489

*CI* confidence interval. *HRT* Hormone Replacement Therapy. Δ = Change. ^a^ Confidence intervals and standard errors for Post-menopause group based on 1000 bootstrap samples

For the pre-menopausal group, step 1’s known predictors accounted for 4% of the variance in sexual functioning, *R*^2^ = 0.04, *F* (2, 281) = 6.22, *p* = 0.002. Step 2, with the YPSQ scores, accounted for an additional 14% of the variance, Δ*R*^2^ = 0.14, *F*Δ (1, 280) = 47.55, *p* < 0.001. The three variables totalled accounted for 18% of the variance, *R*^2^ = 0.18, *F* (3, 280) = 20.68, *p* < 0.001. For the peri-menopausal group, Step 1 was not significant, *R*^2^ = 0.00, *F* (2, 133) = 0.18, *p* = 0.836, but the model was significantly improved by the addition of YPSQ, accounting for a total of 34% of the variance, *R*^2^ = 0.34, *F* (3,132) = 22.56, *p* < 0.001. For the post-menopausal group, psychological/physical problems and HRT at step 1 accounted for a significant 15% of the variance, *R*^2^ = 0.15, *F* (2, 44) = 3.88, *p* = 0.028; Step 2’s inclusion of YPSQ resulted in an increased contribution of 1% of the variance [Δ*R*^2^ = 0.01, Δ*F* (1, 43) = 0.46, *p* < 0.001], but the total model was not significant, *R*^2^ = 0.16, *F* (3, 43) = 2.75, *p* = 0.055. Refer to Table 6.

### Sexual Satisfaction

To test the hypothesis that higher levels of early adaptive schema would be significantly associated with higher levels of sexual satisfaction for each group, a hierarchical MRA was performed using the four YPSQ domains as independent variables. Table 7 shows the means for each group and the variance accounted for by the four domains. The means for each group leaned more towards higher levels of satisfaction reflecting a characteristically assymetrical distribution (Fisher et al., 2011). Table 8 reports the MRA with the four domains, showing the unstandardised (*B*) and standardised (*β*) regression coefficients, and squared semi-partial correlations (*sr*^2^). The four domains were associated with a significant variance in the pre- and peri-menopause groups [*R*^2^ = 0.174, *F* (4, 279) = 14.65, *p* < 0.001; *R*^2^ = 0.57, *F* (4, 131) = 43.69, *p* < 0.001] and was unsignificant for the post-menopause group, *R*^2^ = 0.10, *F* (4, 42) = 1.12, *p* = 0.358. The domains of Connection and Acceptance, and Healthy Autonomy and Performance were both significantly associated with the outcome in the pre-menopause group (*p* = 0.002; *p* = 0.047). The domain of Realistic Standards and Reciprocity was the only significant domain in the peri-menopause group (*p* = 0.007).

**Table 7:** Sexual satisfaction and YPSQ domains

Groups	Mean (*SE*)	*R*^2^	Adj *R*^2^	*p*	Effect
Pre-menopause (*n* = 284)	66.73 (18.74)	0.17	0.16	< 0.001	Medium
Peri-menopause (*n* = 136)	73.49 (13.94)	0.57	0.56	< 0.001	Large
Post-menopause (*n* = 47) ^b^	62.15 (23.73)	0.10	0.01	0.358	NS

^a^Cohen (1962) effect sizes, ^b^bootstrapped, NS = Not significant

**Table 8:** MRA1 sexual satisfaction with four YPSQ domain variables

Groups	*B* [95% CI]	*SE B*	β	*p*
Pre-menopause *R*^2^ = 0.174, *F* (4, 279) = 14.65, *p* < 0.001				
Constant	29.44 [18.69, 40.19]	5.46		< 0.001
Realistic Standards and Reciprocity	3.20 [–1.57, 7.97]	2.42	0.15	0.188
Connection and Acceptance	7.50 [2.74, 12.26]	2.42	0.33	0.002
Healthy Autonomy and Performance	–3.91 [–7.78, –0.05]	1.96	–0.19	0.047
Reasonable Limits	2.35 [–1.48, 6.17]	1.94	0.11	0.228
Peri-menopause *R*^2^ = 0.57, *F* (4, 131) = 43.69, *p* < 0.001				
Constant	19.96 [11.56, 28.35]	4.24		< 0.001
Realistic Standards and Reciprocity	7.99 [2.20, 13.78]	2.93	0.47	0.007
Connection and Acceptance	2.77 [–3.67, 9.20]	3.25	0.16	0.397
Healthy Autonomy and Performance	–0.44 [–5.76, 4.89]	2.69	–0.25	0.872
Reasonable Limits	2.68 [–1.42, 6.77]	2.07	0.17	0.198
Post-menopause^a^ *R*^2^ = 0.10, *F* (4, 42) = 1.12, *p* = 0.358				
Constant	46.71 [2.65, 86.07]	21.11		0.037
Realistic Standards and Reciprocity	–14.51 [–31.62, 8.13]	10.01	–0.42	0.243
Connection and Acceptance	11.72 [–3.61, 28.62]	8.27	0.40	0.233
Healthy Autonomy and Performance	7.23 [–12.00, 21.55]	8.37	0.26	0.884
Reasonable Limits	–1.50 [–12.71, 12.87]	6.47	–0.06	0.660

*CI* confidence interval. ^a^ Confidence intervals and standard errors for Post-menopause group based on 1000 bootstrap samples

To identify whether early adaptive schema were associated with more variance than factors previously shown to predict sexual satisfaction, a two-step hierarchical MRA was utilized. Step 1, entered as a block, included sexual functioning and the presence of a physical and/or psychological disorders. Step 2 added the total YPSQ. For the pre-menopausal group, step1 accounted for 37% of the variance in sexual satisfaction, *R*^2^ = 0.37, *F* (2, 281) = 80.97, *p* < 0.001. Step 2, with the YPSQ, accounted for an additional 2% of the variance, Δ*R*^2^ = 0.02, *F*Δ (1, 280) = 7.79, *p* = 0.006, totalling 38% of the variance, *R*^2^ = 0.38, *F* (3, 280) = 57.89, *p* < 0.001. For the peri-menopausal group, step 1 accounted for 39% of the variance in sexual satisfaction, *R*^2^ = 0.39, *F* (2, 133) = 42.9, *p* < 0.001, and Step 2 significantly improved the model with an additional 23% of the variance accounted for, Δ*R*^2^ = 0.23, Δ*F* (1, 132) = 80.79, *p* < 0.001. The entire model contributed 62% of the variance, *R*^2^ = 0.62, *F* (3,135) = 72.69, *p* < 0.001. For the post-menopausal group, bootstrapped results at Step 1 showed sexual functioning and psychological/medical problems accounted for 53% of the variance, *R*^2^ = 0.53, *F* (2, 44) = 24.95, *p* < 0.001; Step 2’s inclusion of the YPSQ was not significant but the total model retained significance and accounted for 55% of the variance, *R*^2^ = 0.55, *F* (3, 43) = 17.78, *p* < 0.001. Refer to Table 9.

**Table 9:** MRA2 sexual satisfaction: two-step hierarchical multiple regression model

Groups		*B* [95% CI]	*SE B*	*β*	*p*
Pre-menopause	Step 1 *R*^2^ = 0.37 (*p* < 0.001)				
	Constant Sexual Functioning Physical/Psychological Disorder	5.03 [–5.08, 15.14] 2.36 [1.99, 2.73] –3.66 [–7.78, 0.47]	5.14 0.19 2.10	0.60 –0.08	0.328 < 0.001 0.082
	Step 2 Δ*R*^2^ = 0.02 (*p* = 0.006), *R*^2^ = 0.38 (*p* < 0.001)				
	Constant	–4.24 [–16.18, 7.70]	6.07		0.485
	Sexual Functioning Physical/Psychological Disorder	2.14 [1.74, 2.54]	0.20	0.54	< 0.001
		–1.70 [–6.00, 2.60]	2.19	–0.04	0.437
	Total YPSQ	0.87 [0.26, 1.49]	–0.31	0.15	0.006
Peri-menopause	Step 1 *R*^2^ = 0.39 (*p* < 0.001)				
	Constant	24.79 [13.57, 36.02]	5.68		< 0.001
	Sexual Functioning	1.99 [1.54, 2.43]	0.22	0.60	< 0.001
	Physical/Psychological Disorder	–7.60 [–13.36, –1.83]	2.91	–0.18	0.01
	Step 2 Δ*R*^2^ = 0.23 (*p* < 0.001), *R*^2^ = 0.62 (*p* < 0.001)				
	Constant	10.44 {1.02, 19.86]	4.76		0.030
	Sexual Functioning	0.86 [0.43, 1.29]	0.22	0.60	< 0.001
	Physical/Psychological Disorder	–5.54 [–10.12, –0.96]	2.32	–0.13	0.018
	Total YPSQ	0.63 [0.49, 0.77]	0.07	0.59	< 0.001
Post-menopause^a^	Step 1 *R*^2^ = 0.53 (*p* < 0.001)				
	Constant	22.42 [1.30, 46.37]	11.76		< 0.067
	Sexual Functioning	1.90 [1.34, 2.62]	0.27	0.73	0.001
	Physical/Psychological Disorder	–0.50 [–10.60, 9.05]	4.90	–0.01	0.926
	Step 2 Δ*R*^2^ = 0.02 (*p* = 0.150), *R*^2^ = 0.554 (*p* < 0.001)				
	Constant	2.62 [–29.30, 30.54]	14.89		0.849
	Sexual Functioning	1.89 [1.27, 2.34]	0.28	0.73	0.001
	Physical/Psychological Disorder	–3.37 [–13.89, 6.12]	5.20	–0.07	0.521
	Total YPSQ	1.37 [–0.08, 3.10]	0.82	0.16	0.097

*CI* confidence interval. *HRT* Hormone Replacement Therapy. ^a^ Confidence intervals and standard errors for Post-menopause group based on 1000 bootstrap samples

## Discussion

This study aimed to examine the relationship between early adaptive schema and sexual satisfaction and sexual functioning, as key components of sexual wellbeing, on women at different stages of menopause: pre-, peri-, and post-menopause. To our knowledge, this is the first empirical study of the relationship between early adaptive schema on sexual wellbeing in women at different menopausal stages. Interestingly, this study took place during the COVID-19 epidemic. Early research in this area suggests that restrictions relating to COVID-19 resulted in higher rates of sexual dysfunction, especially for women, possibly due to decreased social activities, increased anxiety regarding COVID-19 transmission, and increased environmental stressors regarding unemployment, work stress, and the presence of children in the household (Masoudi et al., 2022).

### Pre-menopausal Group

Higher levels of early adaptive schema were associated with higher sexual functioning and sexual satisfaction in the pre-menopausal group, supporting the first hypothesis for this group. The YPSQ’s level of association with sexual wellbeing in this group was medium (Cohen, 1962) and this differed from the other two groups, supporting the second hypothesis that the relationship with early adaptive schema would vary between groups. This finding supports previous research that found positive sexual schemas were associated with higher sexual functioning (Cyranowski & Andersen, 1998; Peixoto & Nobre, 2020) and that individual constructs of our sexual selves are associated with sexual health (Horley & Clarke, 2016).

The third hypothesis, that the relationship between the early adaptive schema’s influence on sexual functioning and sexual satisfaction will vary based on the menopause status of the group was also supported with the association between theYPSQ domains varying in each group. Within the pre-menopause group, the YPSQ domain of Connection and Acceptance was the significant variable for sexual functioning. Connection and Acceptance includes the early adaptive schema of Emotional Fulfilment and Social belonging, key factors of relationship development. The logic of these connections has been supported by McCool-Myers et al.’s (2018) systematic review of female sexual dysfunction that analysed research across 41 countries and identified relationship dissatisfaction as a consistently significant risk factor (McCool-Myers et al., 2018). When this study’s model for sexual functioning was extended with previously researched predictors, namely physical/psychological disorders and the partner’s sexual functioning status, the previously researched predictors produced a small association, and the model was significantly improved with the addition of early adaptive schema, producing a medium impact on sexual functioning. This outcome reinforced the finding that higher levels of early adaptive schema are associated with higher sexual functioning in pre-menopausal women.

The level of early adaptive schema also had a medium impact on sexual satisfaction in the pre-menopausal group. Connection and Acceptance was the strongest variable, implicating the influence of relationships on sexual satisfaction, and Healthy Autonomy and Performance was also significantly associated with sexual satisfaction in this group. Healthy Autonomy and Performance includes the early adaptive schema of Healthy Boundaries and Stable Attachment. Though research regarding early adaptive schema is still developing, current research on early maladaptive schemas identifies a strong significant relationship between insecure attachment styles in childhood and maladaptive schemas in adulthood, which, in turn predicts insecure relationships (Janovsky et al., 2020; Simard et al., 2011). A similar logic may occur with higher early adaptive schema resulting from secure attachment in childhood that, in turn, may lead to more secure adult relationships and higher relationship satisfaction.

When the known factors for predicting sexual satisfaction in the pre-menopausal group were included, sexual functioning and the presence of physical/psychological disorders, resulted in a large impact on sexual satisfaction. The addition of early adaptive schema produced a small significant positive change. These results suggest that early adaptive schema may be associated with sexual satisfaction predominantly through their association with sexual functioning in the pre-menopausal group.

### Peri-menopausal Group

Higher levels of early adaptive schema were associated with higher levels of sexual functioning within the peri-menopausal group. No domain was significantly strong but the four domains, together, resulted in a large impact. The results supported all three hypotheses in the peri-menopause group. Peri-menopause is a significant physical and psychological transition for women, from reproductive ability to non-ovulation that can last from one to eight years, and is not well understood (Delamater & Santoro, 2018). It may be that many of the women who responded to the survey identified with the symptoms of peri-menopause (for example, irregular menstruation, vasomotor symptoms, and sleep disturbance) rather than fitting the biological definition of peri-menopause that identifies decreased reproductive hormones (Delamater & Santoro, 2018). Regardless, higher levels of early adaptive schema may act as a protective factor for women’s sexual functioning during this period of uncertainty and physical change. The strong relationship between early adaptive schema and sexual functioning in the peri-menopause group was significant, in addition to the relationship between physical/psychological disorders and partner’s sexual dysfunction, confirming the importance of considering early adaptive schema’s relationship with sexual functioning for peri-menopausal women in a therapeutic context.

As with sexual functioning, high levels of early adaptive schema had a large significant relationship with sexual satisfaction. In support of hypothesis three, that the groups’ sexual wellbeing will be associated with different early adaptive schema, the domain of Realistic Standards and Reciprocity had a significant independent relationship with sexual satisfaction in the peri-menopause group. This early adaptive schema includes Empathatic Consideration and Self-Compassion schemas. Sexual satisfaction, as measured by the NSSS, contains two sub-scales: an ego-focused subscale and a partner and activity-focused subscale (Štulhofer et al., 2010). The link between Empathatic Consideration and sexual satisfaction may be explained by an individual’s ability to be a considerate partner during sexual interaction which then, considering the reciprocal nature of sexual intercourse, has a direct effect on their own sexual satisfaction (McCabe et al., 2010).

The large positive relationship between early adaptive schema and sexual satisfaction remained in addition to the variance accounted for by sexual functioning and physical/psychological disorders. In this group, early adaptive schema may be related to sexual satisfaction directly, and indirectly through its impact on sexual functioning. The strong relationship between early adaptive schema and sexual wellbeing in the peri-menopausal group supports Videler et al. (2020) theory that early adaptive schema are more active and early maladaptive schema less active in middle age due to the accumulation of knowledge and increased coping strategies (Videler et al., 2020). The result reinforces the potential value of using early adaptive schema in conceptualisation and intervention for both sexual dysfunctions and sexual satisfaction in women who endorse peri-menopausal symptoms.

### Post-menopausal Group

Levels of early adaptive schema were not found to relate to sexual functioning or sexual satisfaction for the post-menopausal group, negating the hypotheses for this group. While the small sample size may have limited the power to find significant associations, the known predictors accounted for significant variance for both sexual functioning and sexual satisfaction. The lack of association between early adaptive schema and sexual wellbeing in the post-menopause group supports previous findings that this group’s sexual wellbeing is significantly impacted by physiological changes relating to menopause, namely hormonal and age-related physiological changes, and the psychological effects related to aging and age-related disorders (Caruso et al., 2016; Simon et al., 2018). The results indicate that the relationship between early adaptive schema and sexual functioning, and between early adaptive schema and sexual satisfaction, reduces considerably post-menopause.

### Theoretical Links

This study strengthens the rationale for the focus on cognitions when treating women with sexual dysfunctions (Peixoto & Nobre, 2020; Stephenson & Meston, 2016) and contributes to the early, but growing, body of evidence supporting the use of early adaptive schema in cognitive therapy. The results demonstrated that early adaptive schema are associated with sexual functioning and sexual satisfaction, reinforcing the view that specific cognitions relating to early adaptive schema may play an important part in the treatment of sexual dysfunctions, and the promotion of sexual satisfaction, especially at pre- and peri-menopause. The lack of association between early adaptive schema and sexual wellbeing in the post-menopause group does not support Socioemotional Selectivity Theory’s “positivity effect” where older adults veer towards processing positive information and avoid negative information to enhance their mood and wellbeing (Carstensen et al., 2003). The outcome may be due to the low power of the group, limiting the study’s ability to find significant results, or the increased age-related biological barriers to sexual functioning and sexual satisfaction that lead to changed goals and priorities, lessening the influence of sexual wellbeing to overall wellbeing in older age (Neto & Pinto, 2013).

The varying levels of associations of early adaptive schema on sexual wellbeing (medium for pre-menopause, large for peri-menopause, and non-significant for post-menopause) support a theory that Videler et al. (2020) proposed when exploring the use of early adaptive schema in the treatment of older adults with personality disorders. Though Videler et al. (2020) accepted the limited research on the stability of early maladaptive schema and early adaptive schema across the lifespan, they posited that early adaptive schema may be more activated and utilised in early and middle adulthood, but initiated less in older adulthood due to the predominance of maladaptive schema used to cope with older-age life stressors, such as increased physical and psychological disorders, death of loved ones, and reduced autonomy (Videler et al., 2020). Therefore, though early adaptive schema levels were not associated with sexual wellbeing in the small group of post-menopause women in the study, integrating early adaptive schema in the treatment of older, post-menopausal women, may re-awaken the healthy adult mode that dominated in their early- to middle-age and enable the development of protective mechanisms against negative views of the aging self and their changing world (Videler et al., 2020).

### Limitations

This study was limited by the use of online self-reports which, though producing an avenue for anonymity, may increase misrepresentation and self-selection bias. The self-selection bias may have affected the post-menopause group particularly, with women who were not sexually active avoiding participation. Using convenience samples sourced from social media and snowballing limits accessibility to the study to more homogenous groups and the generalizability of the results, especially to women in lower socio economic situations. The questionnaire was in English and though the majority of participants identified English-speaking countries as their residence, and the authors believed the use of English would self-select participation, some participants with lower levels of English proficiency may have proceeded with the survey and faced difficulty interpreting the questions. The study itself includes subjectivity within the variables, for example the idiosyncrasy of evaluating sexual satisfaction and conceptualizing sexual wellbeing, in the absence of an agreed definition, as sexual functioning and sexual satisfaction. The scale used to measure sexual function, the FSFI, contained a subset of sexual satisfaction as a sexual dysfunction which produced an overlap with the scale used to assess sexual satisfaction (the NSSS). Both scales have limitations associated with participants not engaged with partnered sex, resulting in skewed results (Meston et al., 2020; Štulhofer et al., 2010).

The study relied on people categorizing themselves as being in pre-, peri-, or post-menopause that may have affected the self-selection of peri-menopause due to the difficulty of determining the classification of this group without a biological hormone test. This resulted in a low mean age for the group (37 years) and limits the application of the findings. Though there are medical definitions for these classifications, there are misconceptions regarding their description, and additionally, the symptoms of peri- and post-menopause can be subdued by hormone treatment (Nazarpour et al., 2016; Simon et al., 2018). For example, women on interuterine devices that reduce or eliminate menstruation may be unaware of their menopause status. For these reasons, the authors decided against using an age limitation for menopause status.

Combining physical/psychological disorders in the known factors precluded the analysis of these variables independently. Additionally, in the post-menopausal group, the sample size limited the number of variables in the regression analysis so that the variables chosen were based on research, frequency, and association with the outcome variable, resulting in a predominance of biopsychological factors. The measures of partner’s sexual dysfunction, HRT, and the presence of physical/psychological disorders was limited by respondent knowledge, the use of questions rather than valid and reliable scales, and the combination of variables, for example, combining yes with unsure/maybe responses. Lastly, the low number of participants identifying as post-menopausal resulted in low power that limited the ability to identify small-medium associations.

Women face various biopsychosocial life-stressors, both similar and distinct to men, that align with their stages of menopause: pre-, peri-, and post-menopause. This study supports the integration of early adaptive schema in the biopsychosocial treatment of sexual dysfunctions for women and to promote sexual wellbeing for women at pre- and peri-menopause. Further research is needed to validate these results with more empirical studies, especially for post-menopause women, to identify relationships between specific early adaptive schema and sexual wellbeing in the support of therapeutic interventions, and to determine whether early adaptive schema treatment is beneficial independently or in association with other biological and psychological treatments.

## Data Availability

The data that support the findings of this study are available from the corresponding author, AA, upon reasonable request.

## Appendix

Tables 10, 11, 12 and 13

**Table 10:** Correlations between sexual functioning and YPSQ four domains

	Group	Total sexual functioning	Realistic standards	Connection & acceptance	Healthy autonomy	Realistic limits
Total Sexual Functioning	Pre-menopause	1.000	0.363	0.382	0.310	0.346
	Peri-menopause	1.000	0.566	0.560	0.569	0.497
	Post-menlopause	1.000	–0.063	0.106	0.050	–0.041
Realistic Standards	Pre-menopause	0.363	1.000	0.840	0.777	0.760
	Peri-menopause	0.566	1.000	0.928	0.918	0.868
	Post-menlopause	–0.063	1.000	0.757	0.788	0.706
Connection & Acceptance	Pre-menopause	0.382	0.840	1.000	0.757	0.744
	Peri-menopause	0.560	0.928	1.000	0.919	0.899
	Post-menlopause	0.106	0.757	1.000	0.649	0.617
Healthy Autonomy	Pre-menopause	0.310	0.777	0.757	1.000	0.756
	Peri-menopause	0.569	0.918	0.919	1.000	0.850
	Post-menlopause	0.050	0.788	0.649	1.000	0.811
Realistic Limits	Pre-menopause	0.346	0.760	0.744	0.756	1.000
	Peri-menopause	0.497	0.868	0.899	0.850	1.000
	Post-menlopause	–0.041	0.706	0.617	0.811	1.000

**Table 11:** Correlations between Sexual Functioning, Physical and Psychological Problems, Sexual Function Disorder, and Total YPSQ

	Menopause group	Total sexual functioning	Physical/ Psychological problems	Sexual function disorder	HRT	Total YPSQ
Total Sexual Functioning	Pre-	1.000	–0.054	0.203		0.384
	Peri-	1.000	0.006	–0.052		0.565
	Post-	1.000	0.031		–0.386	0.020
Physical & Psychological Problems	Pre-	–0.054	1.000	–0.105		–0.316
	Peri-	0.006	1.000	–0.047		–0.082
	Post-	0.031	1.000		–0.004	0.366
Sexual Function Disorder	Pre-	0.203	–0.105	1.000		0.104
	Peri-	–0.052	–0.047	1.000		0.141
	Post-	–0.386	–0.004		1.000	0.221
Total YPSQ	Pre-	0.384	–0.316	0.104		1.000
	Peri-	0.565	–0.082	0.141		1.000
	Post-	0.020	0.366		0.221	1.000

*HRT* Hormone Replacement Treatment – Post-menopause group only

**Table 12:** Correlations between sexual satisfaction and YPSQ four domains

	Group	Total sexual satisfaction	Realistic standards	Connection & acceptance	Healthy autonomy	Realistic limits
Total Sexual Satisfaction	Pre-menopause	1.000	0.365	0.397	0.261	0.329
	Peri-menopause	1.000	0.746	0.728	0.696	0.704
	Post-menlopause	1.000	0.049	0.215	0.145	0.106
Realistic Standards	Pre-menopause	0.365	1.000	0.840	0.777	0.760
	Peri-menopause	0.746	1.000	0.927	0.913	0.869
	Post-menlopause	0.049	1.000	0.757	0.788	0.706
Connection & Acceptance	Pre-menopause	0.397	0.840	1.000	0.757	0.744
	Peri-menopause	0.728	0.927	1.000	0.916	0.898
	Post-menlopause	0.215	0.757	1.000	0.649	0.617
Healthy Autonomy	Pre-menopause	0.261	0.777	0.757	1.000	0.756
	Peri-menopause	0.696	0.913	0.916	1.000	0.840
	Post-menlopause	0.145	0.788	0.649	1.000	0.811
Realistic Limits	Pre-menopause	0.329	0.760	0.744	0.756	1.000
	Peri-menopause	0.704	0.869	0.898	0.840	1.000
	Post-menlopause	0.106	0.706	0.757	0.811	1.000

**Table 13:** Correlations between sexual satisfaction, sexual functioning, physical and psychological problems, and total YPSQ

	Menopause group	Total sexual satisfaction	Sexual functioning	Physical/Psychological problems	Total YPSQ
Total Sexual Satisfaction	Pre-	1.000	0.599	–0.115	0.369
	Peri-	1.000	0.601	–0.179	0.751
	Post-	1.000	0.729	0.012	0.150
Sexual Functioning	Pre-	0.599	1.000	–0.054	0.384
	Peri-	0.601	1.000	–0.004	0.576
	Post-	0.729	1.000	0.031	0.020
Physical & Psychological Problems	Pre-	–0.115	–0.054	1.000	–0.316
	Peri-	–0.179	–0.004	1.000	–0.083
	Post-	0.012	0.031	1.000	0.366
Total YPSQ	Pre-	0.369	0.384	–0.316	1.000
	Peri-	0.751	0.576	–0.083	1.000
	Post-	0.150	0.020	0.366	1.000

**Table 14:** Correlations between sexual functioning, partner sexual functioning, hormone medication use, and physical and psychological problems in the post-menopause group

	Partner Sexual Dysfunction	Physical & Psychological Problems	Hormone Medication Use	Sexual Functioning
Partner Sexual Dysfunction	-	-0.318	-0.030	0.232
Physical & Psychological Problems		-	-0.004	0.031
Hormone Medication Use			-	-0.386
Sexual Functioning				-
